# Loss of nidogen-1 causes lung basement membrane defects and increased metastasis

**DOI:** 10.3389/fimmu.2025.1598547

**Published:** 2025-10-16

**Authors:** Tian Xia, Kamilla W. Zornhagen, Ilkka Miinalainen, Lilach Abramovitz, Chris D. Madsen, Monica Nicolau, Alejandro E. Mayorca-Guiliani, Neta Erez, Janine T. Erler

**Affiliations:** ^1^ Biotech Research and Innovation Centre (BRIC), University of Copenhagen (UCPH), Copenhagen, Denmark; ^2^ Electron Microscopy Core Facility, University of Oulu, Oulu, Finland; ^3^ Department of Pathology, Gray Faculty of Medical & Health Sciences, Tel Aviv University, Tel Aviv, Israel; ^4^ Division of Translational Cancer Research, Lund University, Lund, Sweden

**Keywords:** nidogen-1, basement membrane, lung, cancer, metastasis

## Abstract

Metastasis is the most common cause of cancer patient deaths. It is a complex process strongly influenced by the extracellular matrix (ECM). A mass spectrometry analysis comparing ECM proteins from healthy mouse lungs versus metastatic lungs has previously been performed, and the basement membrane (BM) component nidogen-1 has been identified to be one of the most downregulated ECM proteins in metastatic lungs. Here, we investigated the role of stromal cell-derived nidogen-1 in metastasis. We found that nidogen-1 is expressed by fibroblasts but not cancer cells, and nidogen-1 is downregulated in breast tumors compared to healthy mammary gland. Using the HCmel12 melanoma model, we found that loss of stromal nidogen-1 causes increased lung metastasis. Using electron microscopy, we found that nidogen-1 knockout mice have defects in the lung alveoli, such as fragmented endothelium, poorly defined BM, and enlarged interstitium. This suggests that loss of nidogen-1 may cause BM defects, which compromise its barrier function, thereby increasing the ability of cancer cells to extravasate and colonize the lungs. Our findings provide novel insight into cancer-stromal interplay and the role of nidogen-1 at the metastatic niche.

## 1 Introduction

Metastasis is the most common cause of death for patients with solid malignant tumors (1). The major steps of metastasis, also known as the invasion-metastasis cascade, include local invasion, intravasation, extravasation, and metastatic colonization (2, 3). These steps require cancer cells being able to breach mechanical barriers between tissues, such as basement membranes (BMs), which is a specialized form of extracellular matrix (ECM) containing laminins, collagens, perlecans and nidogens (1, 4).

It has been shown that metastatic potential of cancer cells correlates with their ability to degrade BM components (5), and that invasive tumors are surrounded by fragmented and disrupted BMs while their benign counterparts are surrounded by intact BMs (6). This suggests that BMs are crucial structural ECM barriers inhibiting cancer metastasis.

In our previous studies, we performed a mass spectrometry analysis comparing ECM proteins in decellularized tissues from healthy lungs versus metastatic lungs from mice bearing orthotopic breast cancer tumors, and identified nidogen-1 as one of the most downregulated ECM components in metastatic lungs (7). In addition, in that study, we also observed discontinuous nidogen-1 coverage of the BMs at the invasive tumor front. This led us to hypothesize that loss of nidogen-1 in the stromal compartment may promote cancer cell invasion and metastasis (7).

Here, we investigate the role of stromal nidogen-1 in cancer metastasis using nidogen-1 knockout mice. Our findings uncover a novel role of stromal nidogen-1 in the regulation of cancer progression and identify lung BM defects upon loss of nidogen-1 that enable formation of metastases. Our studies therefore uncover novel functions of nidogen-1 in regulating cancer progression and highlight the roles of stromal cell-derived nidogen-1.

## 2 Materials and methods

### 2.1 Cell culture

4T1 and B16 cells were cultured in DMEM (Gibco) with 10% FBS. HCmel12 cells were cultured in DMEM with 20% FBS. Cancer-associated fibroblasts (CAFs) was cultured in DMEM with 10% FBS and 1% Insulin-Transferrin-Selenium (100X, Gibco).

### 2.2 CRISPR knockout cell line generation

The generation of nidogen-1 CRISPR knockout cell lines was performed according to published protocols (8). sgRNA for CRISPR was designed using the DESKGEN cloud platform (9). Two sgRNA oligos 5’- CACCGCTGCCATCTGAATAATGAA -3’ and 5’- AAACTTCATTATTCAGATGGCAGC -3’ targeting nidogen-1 were cloned into the vector SpCas9-2A-Puro V2.0 8. After transfection, single cell isolation was performed. Knockout clones were confirmed by Sanger sequencing of the genomic DNA.

### 2.3 Animal studies

All animal experiments were approved and conducted according to the regulations of The Animal Experiments Inspectorate, Danish Veterinary and Food Administration, with license numbers 2012-15-2934–00222 and 2017-15-0201-01265, as well as regulations from the Department of Experimental Medicine, University of Copenhagen. We obtained the nidogen-1 knockout mouse as a generous gift from Prof. Thomas Krieg at the University Hospital of Cologne (10). The αSMA::RPF transgenic mouse was published previously (11). The PyMT-Col1α::YFP transgenic mice were generated and maintained by the Erez Lab (Raz Y. and Cohen N. et al., under revision). Other wildtype mice were ordered from Taconic Biosciences.

For the tumor-fat pad junction staining experiments, 1,000,000 4T1 H2B GFP cells were injected into the mammary fat pad of 10-week old BALB/c mice. Primary tumors together with mammary fat pads were resected 10 days after injection. For αSMA+ stromal cell and 4T1 FACS sorting experiments, 1,000,000 4T1 H2B GFP cells were injected into the mammary fat pad of 16-week old αSMA::RPF transgenic mice. Primary tumors together with mammary fat pads were resected 8 days after injection. For melanoma orthotopic models, 200,000 HCmel12 cells were injected subcutaneously into 10-18-week old mice. Humane endpoint was defined when primary tumor reached 10mm diameter. For melanoma experimental metastasis models, 200,000 HCmel12 cells were injected into the lateral tail veins of 9-11-week old mice. Mice were euthanized 4 weeks after injection.

### 2.4 Isolation of cells from fresh tissues by Fluorescence Activated Cell Sorting

The isolation of normal mammary gland fibroblasts (NMFs) and cancer-associated fibroblasts (CAFs) from the PyMT model, and healthy lung fibroblasts and metastatic lung fibroblasts from the BALB/c model were performed as previously described (12). This reference (12) published in the Journal of Visualized Experiments (JoVE) is a detailed step-by-step protocol methodology paper (with videos and graphs).

**Figure 1:**
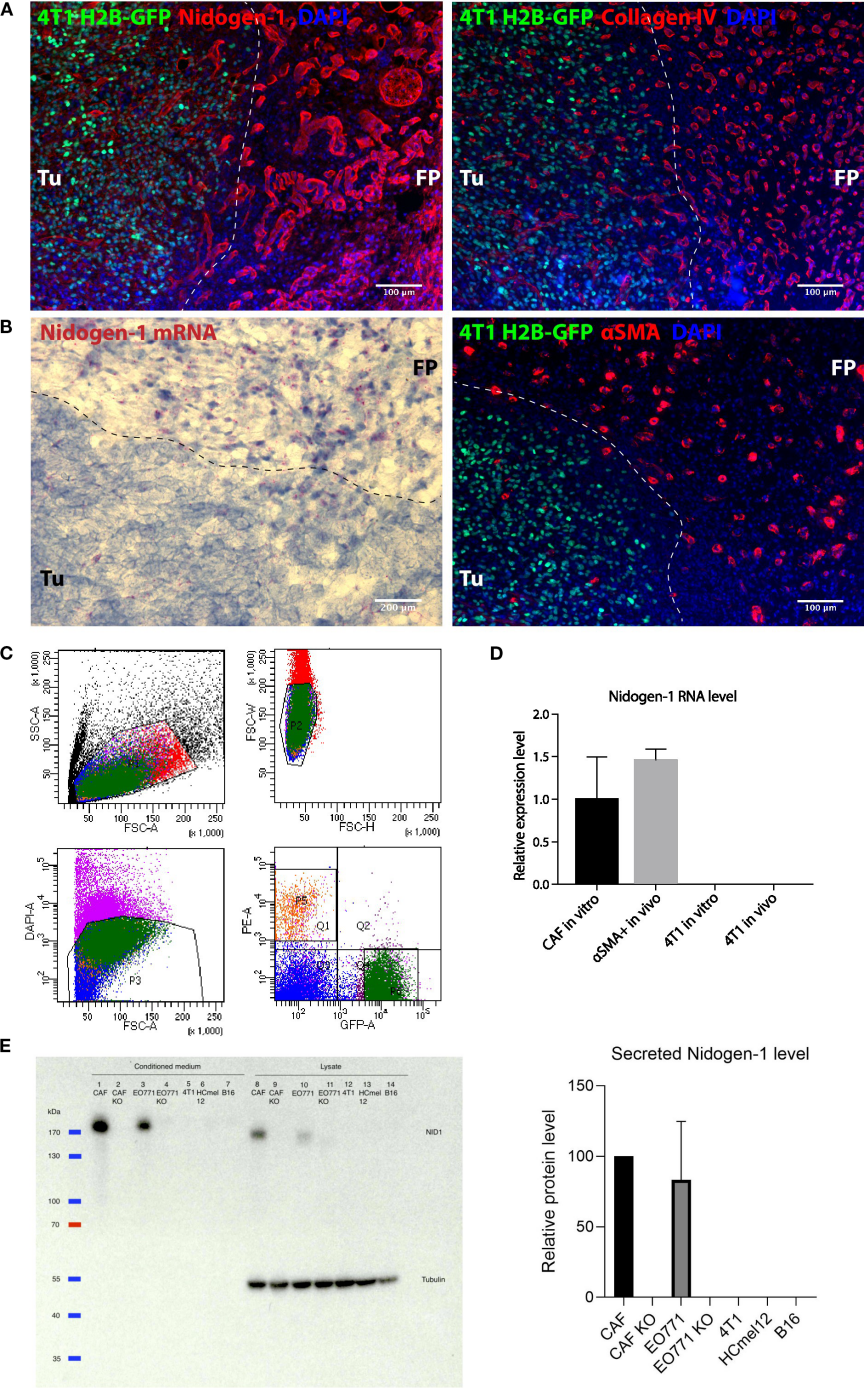
Nidogen-1 is expressed by fibroblasts but not by cancer cells. **(A)** Immunofluorescent images of a fat pad bearing a primary tumor. **(B)** Left: RNAscope image showing nidogen-1 mRNA (pink dots); right: immunofluorescent image of a fat pad bearing a primary tumor. **(C)** Fluorescence Assisted Cell Sorting (FACS) sorting panels. Q1 represents αSMA::RFP, and P6 represents 4T1 H2B-GFP. **(D)** qPCR quantification of nidogen-1 levels. **(E)** Left: western blot (WB) of nidogen-1; right: quantification of the western blot signal in the conditioned medium of three biological repeats (CAF normalized to be 100). Lane1: CAF, 2: CAF KO, 3: EO771, 4: EO771 KO, 5: 4T1, 6: HCmel12, 7: B16, from conditioned medium. Lane8: CAF, 9: CAF KO, 10: EO771, 11: EO771 KO, 12: 4T1, 13: HCmel12, 14: B16, from cell lysates. Full western blot films and more information is in **Supplementary Figure 2A, B**, **(C)** Note: EO771 cell line was not used in this manuscript. For transparency, we did not cut this WB membrane and therefore EO771 line was kept here. “KO” refers to nidogen-1 knockout using CRISPR technology. Tu, tumor region; FP, fat pad region. N numbers in this figure: for **Figures 1A, B**, 6 mice were examined (n=6). Representative picture shown; for **Figure 1C**, for both the *in vivo* and *in vitro* experiments, three mice were used for each group (n=3 per group); for **Figure 1D**, three independent biological repeats were performed (n=3).

Cell exclusion was performed using the CD326 (EpCAM) antibody (clone G8.8, eBioscience) and the CD45 antibody (clone 30-F11, eBioscience). PDGFRα antibody used was clone APA5 (eBioscience). The isolation of GFP+ and RFP+ cells from the αSMA::RPF model was performed using the gentleMACS Dissociator following Miltenyi Biotec’s instructions. We have also included detailed control data for tumor cells control (**Supplementary Figure 6**), dsRed channel control (**Supplementary Figure 7**), and GFP channel control (**Supplementary Figure 8**). In addition, detailed view of the FACS experiment in **Figure 1C** is also included (**Supplementary Figure 9**).

### 2.5 RNA isolation and qPCR

RNA from sorted cells was isolated using the RNeasy Micro Kit (Qiagen), followed by cDNA synthesis using the iScript cDNA Synthesis Kit (Bio-Rad). qPCR was performed using the LightCycler 480 SYBR system (Roche). Nidogen-1 oligos were 5’-GTATCCCCCTCCCTGGAACT-3’ and 5’-TCGCTCATGGCGATGATACC-3’. Col1α oligos were 5’-TGTGTTCCCTACTCAGCCGTCT-3’ and 5’-CTCGCTTCCGTACTCGAACG-3’. αSMA oligos were 5’-AGCCAGTCGCTGTCAGGAA-3’ and 5’-CGAAGCCGGCCTTACAGA-3’.

For the qPCR analysis in **Figure 1**, we first calculate the mean CT from raw CT, and then deducted b-actin value which gives the dCT value. And then we calculated the 2^-dCT*100 value. In the end, we normalize CAF *in vitro* = 1.

**Figure 2:**
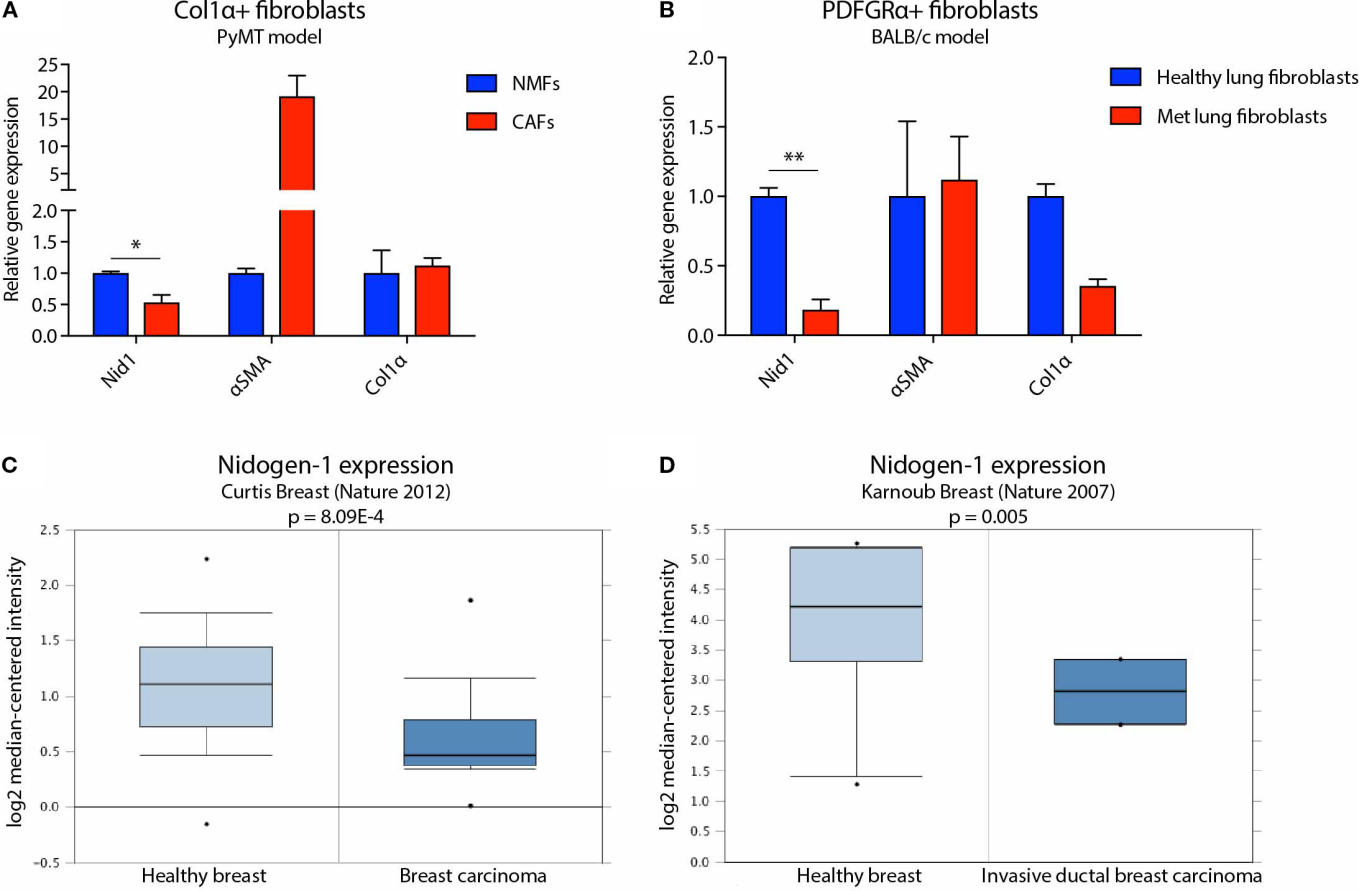
Nidogen-1 expression is downregulated in breast tumors compared to healthy mammary gland. **(A)** qPCR quantification of nidogen-1, αSMA, and Col1α mRNA level in Col1α^+^ fibroblasts. NMF, normal mammary gland fibroblast; CAF, cancer associated fibroblast. n = 4 for each group (pooled as two repeats). **(B)** qPCR quantification of nidogen-1, αSMA, and Col1α mRNA level in PDGFRα^+^ fibroblasts. n = 6 for each group (pooled as two repeats). Met, metastatic. **(C)** Nidogen-1 mRNA level in the Curtis clinical dataset. n = 2,136 in total. **(D)** Nidogen-1 mRNA level in the Karnoub clinical dataset. n = 22 in total. *p < 0.05, **p< 0.01 by two-tailed Student’s *t*-test. Col1α here refers to Col1α1 (HGNC ID HGNC:2197).

For qPCR analysis in **Figure 2**, mGUS was used as a housekeeping gene. We first calculate the mean CT from raw CT, and then deducted mGUS value which gives the dCT value. And then we calculated the 2^-dCT*100 value. Then we normalized NMF or healthy lung fibroblast as 1 and normalized the rest values accordingly.

Different housekeeping genes were used because these two experiments were performed in two different labs (Erler Lab in University of Copenhagen and Erez Lab in Tel Aviv University). We acknowledge that in an ideal situation the same housekeeping gene should be used within the same study.

### 2.6 Western blots

1,000,000 cells were seeded in a 10-cm dish in their normal growth medium. The day after, normal medium was switch to serum free medium. 24hr after, conditioned medium (CM) was collected, filtered through a 0.45 μm filter, and concentrated with Vivaspin 6 centrifugal concentrators (Sartorius). RIPA buffer was used to prepare cell lysates. Protein concentration was measured and adjusted using Bradford assay. For each condition, equal amount protein was loaded for western blot. Antibody against nidogen-1 was AP02274SU-N from Acris. Quantification of western blot results was performed using the Plot Lanes function of the Fiji software. In **Supplementary Figures 2A, B** we provided the full films of the western blots with protein size ladders labeled on 2b.

### 2.7 Quantification of lung metastasis

The left lung lobes were fixed in 10% neutral buffered formalin solution (Sigma) overnight at room temperature before stored in 70% ethanol at 4 °C. Lung lobes were then embedded in paraffin and sectioned with maximized surface area at 3 μm thickness. 6-step sections were performed, with 500 μm distance between step 3 and 4, and 100 μm distance between other steps. Haematoxylin and eosin (H&E) staining were performed to score lung metastatic tumors. For each mouse, the final lung metastasis foci number is the average number of the 6 sections scored.

### 2.8 Tissue processing, immunofluorescence and RNA *in situ* hybridization

For the tumor-fat pad junction staining, samples were preserved in 30% sucrose at 4°C overnight before embedding with OCT and snap frozen using dry ice. Samples were stored at -80°C. Tissue blocks were then cryosectioned at 6 μm thickness.

For immunofluorescent staining, frozen sections were first incubated at 37°C for 10 min, then fixed in 4% paraformaldehyde solution. Slides were then permeabilize using TBST (0.05% tween-20) followed by blocking with 5% normal serum from the host of the secondary antibody. Primary antibodies against nidogen-1 (1:100, Acris, AP02274SU-N), collagen IV (1:100, Millipore, AB756P) and αSMA (1:100, Sigma, C6198) was incubated overnight at 4 °C. Appropriate secondary antibodies (Alexa Fluor) were incubated for 1 hr at room temperature.

RNA *in situ* hybridization was performed using RNAscope 2.5 HD Detection Reagents-RED system following manufacturer’s protocol (Doc. Num. 320534 and 322350-USM). Nidogen-1 probe number is REF482541.

### 2.9 Electron microscopy

Transmission Electron Microscopy (TEM) was performed at the Oulu EM Core Facility. Lung samples were fixed in 0.1 M phosphate buffer containing 1% glutaraldehyde and 4% formaldehyde. Samples were then post-fixed in 1% osmium tetroxide, dehydrated in acetone and embedded using the LX-112 kit (Ladd Research). Thin sections were cut at 70nm using the Ultracut UCT ultramicrotome (Leica), stained in uranyl acetate and lead citrate and examined in the Tecnai G2 Spirit TEM microscope (FEI). Images were captured by the Quemesa camera and analyzed using the iTEM software (Olympus).

## 3 Results

### 3.1 Nidogen-1 is expressed by fibroblasts but not cancer cells

We previously identified nidogen-1 as strongly downregulated in metastases of the lungs compared to healthy lung tissue, using mass spectrometry analysis of decellularized organs obtained from the 4T1 syngeneic mouse model of breast cancer (7). It has previously been shown that nidogen-1 is expressed in mammary mesenchymal and myoepithelial cells, but not in epithelial cells (13). In addition, most fibroblast cell lines express nidogen-1 (13). We therefore initially focused on breast cancer and examined the nidogen-1 expression pattern in the 4T1 mouse model of breast cancer.

We injected 4T1 breast cancer cells expressing histone H2B-GFP into the mammary fat pad of syngeneic BALB/c mice and stained the border region of the 4T1 tumors and healthy fat pad with both nidogen-1 and collagen IV antibodies to visualize the BM (**Figure 1A**). Both nidogen-1 and collagen IV were present at the tumor region and the healthy fat pad region. The morphology of the stained structures suggests that both nidogen-1 and collagen IV are located at the BM surrounding vessels and mammary ducts (**Figure 1A**). However, nidogen-1 staining was stronger in the healthy fat pad region as compared to the tumor region. Since this is a staining of one single slide containing border regions of both tumor and healthy fat pad tissues and both regions on the same slide were treated with the same antibodies, incubation conditions, microscopy settings, we could reach the conclusion that nidogen-1 staining was stronger in the healthy fat pad region as compared to the tumor region.

We have also performed CD31 and nidogen-1 co-localization immunofluorescent staining in 4T1 primary tumor samples (**Supplementary Figure 3**), and the co-localization results supports nidogen-1 is located at the BM surrounding vessels.

In addition, we provided deeper characterization of nidogen-1 from higher magnification immunofluorescent staining images showing detailed views of nidogen-1 in primary 4T1 tumor in fat pad area (**Supplementary Figure 4**) and in lung metastasis of 4T1 tumor area (**Supplementary Figure 5**).

To investigate which cells were producing nidogen-1, we used RNAScope technology to visualize nidogen-1 mRNAs in the border region of the 4T1 tumor and the healthy fat pad. We found that the majority of nidogen-1 mRNA (pink dot) is present in the healthy fat pad region and potentially associate with the expression pattern of αSMA (**Figure 1B**). These findings suggested that nidogen-1 is expressed by αSMA expressing cells and not the 4T1 cancer cells.

To confirm that nidogen-1 is expressed by αSMA positive cells and not 4T1 cancer cells, we injected H2B-GFP labelled 4T1 cells into αSMA::RPF transgenic mice (11). We then resected the primary tumor, performed single cell isolation, and used FACS to sort out GFP + 4T1 cells and RFP+ cells (containing fibroblasts, pericytes and myoepithelial cells) (**Figure 1C**). To quantify the mRNA levels of nidogen-1 in the *ex vivo* sorted out cells, we performed qPCR analysis. We also compared the nidogen-1 expression to *in vitro* cultured 4T1 and CAFs, which are major αSMA expressing cells present in tumors. The results indicated that nidogen-1 was expressed in αSMA+ cells and confirmed that 4T1 cells do not express nidogen-1 in tumors (**Figures 1C, D**).

To further investigate these findings at the protein level, we performed western blot analysis of nidogen-1 protein expression in 4T1 breast cancer cells and CAFs. In order to test the generalizability of the findings, we also included two melanoma cell lines, HCmel12 and B16F10. Analysis of the results confirmed that nidogen-1 is expressed in CAFs but not in 4T1, HCmel12, and B16F10 cancer cells (**Figure 1E**).

In order to confirm the specificity of the nidogen-1 antibody (Acris, AP02274SU-N), we utilized CRISPR to generate CAF nidogen-1 knockout cells. Western blot analysis showed an absence of nidogen-1 expression in the knockout cells (**Figure 1E**), validating our findings. Taken together, these findings suggest that nidogen-1 is highly expressed in CAFs but not in cancer cells.

### 3.2 Nidogen-1 is downregulated in breast tumors compared to healthy mammary gland

We previously identified nidogen-1 as being downregulated in 4T1 breast tumors compared with healthy mammary fat pad (7). We therefore investigated whether CAFs express less nidogen-1 than normal fibroblasts. We examined this using two different mouse models of breast cancer (**Figures 2A, B**).

The first model was based on the PyMT transgenic breast cancer model, which develops spontaneous tumors. These mice were manipulated to express YFP when the collagen α1 gene is expressed, which predominantly occurs in CAFs (22). In this first model, we compared Col1α+ fibroblasts from healthy Col1α::YFP transgenic mice with fibroblasts from tumor bearing Col1α::YFP transgenic MMTV-PyMT mice (**Figure 2A**). We FACS sorted out YFP+ cells and performed qPCR analysis. We found that CAFs purified from the PyMT mice express 50% lower levels of nidogen-1 compared to fibroblasts isolated from normal mammary glands (**Figure 2A**). Our data also confirm αSMA as a marker for CAFs (14).

We then focused our attention to the metastatic site and used tail vein injection of cells to study the colonization and outgrowth of metastases, and isolate CAFs for analysis of nidogen-1 expression. In this second model, we performed tail vein injection of 4T1 cells into BALB/c mice and compared fibroblasts from healthy and metastatic lungs. Here, we FACS sorted PDGFRα+ fibroblasts and performed qPCR analysis. PDGFRα has been shown to be broadly expressed in normal and neoplastic fibroblasts (15). We have also shown that 4T1 cells do not express PDGFRα (**Supplementary Figure 10**). We found that CAFs from metastatic lungs express 80% lower levels of nidogen-1 compared to healthy lung fibroblasts (**Figure 2B**). These data suggest that fibroblasts in the primary tumor and in metastatic lung foci express less nidogen-1 as compared to the healthy tissue. The data is consistent with our previous mass spectrometry findings that nidogen-1 is slightly downregulated in the primary breast cancer while substantially reduced in the metastatic lungs (7).

In order to test the clinical relevance of our findings, we probed human cancer data sets available from the Oncomine database (16). We found that in both the Curtis Breast dataset (17) and the Karnoub Breast dataset (18), nidogen-1 is downregulated in breast tumors compared to healthy breast tissues (**Figures 2C, D**). Taken together, these findings suggest that nidogen-1 expression is reduced in CAFs in breast tumors compared with healthy tissue.

### 3.3 Stromal-derived nidogen-1 is required to inhibit metastatic tumor establishment

**Figure 3:**
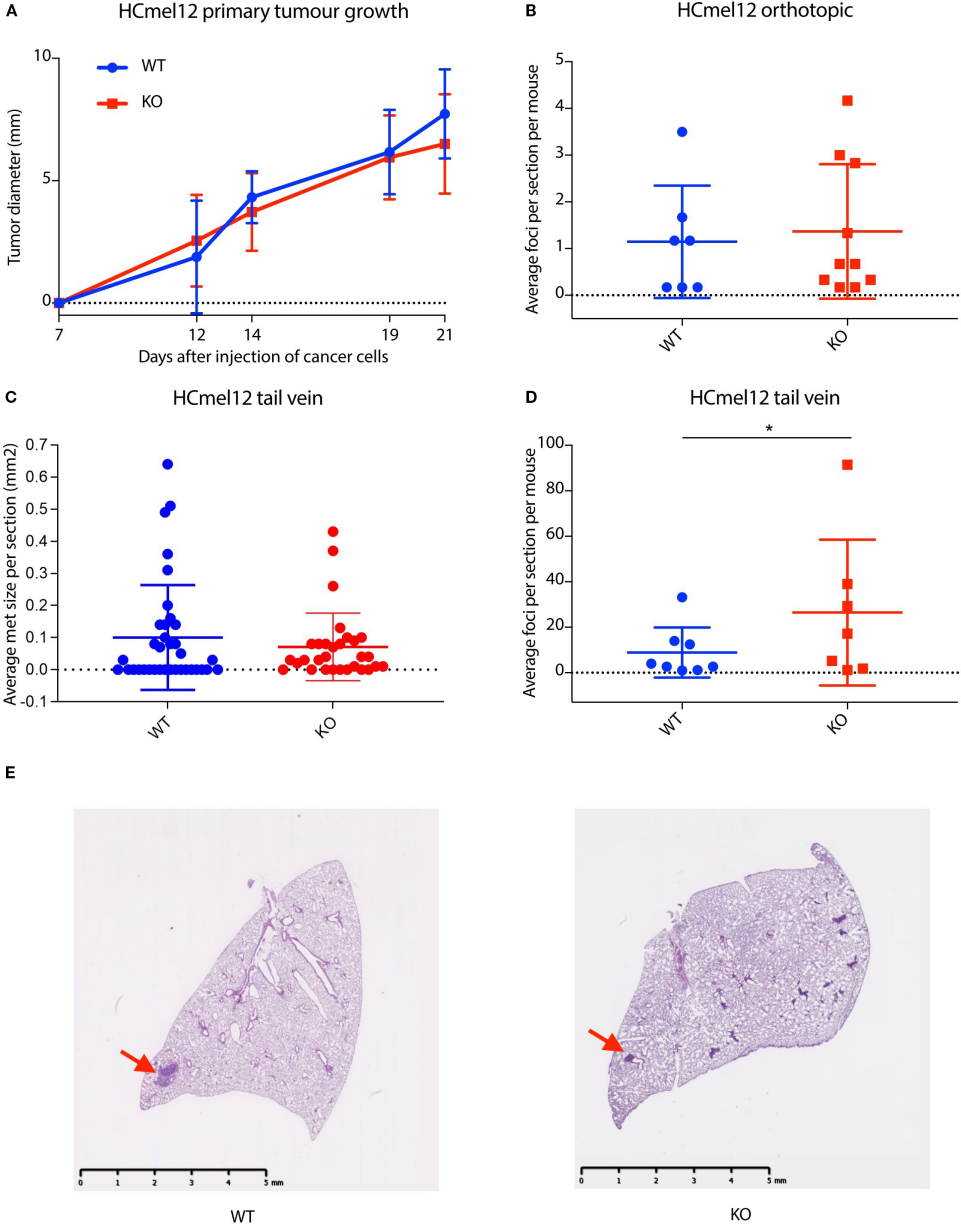
Stromal-derived nidogen-1 is required to inhibit metastatic tumor establishment. **(A)** Primary tumor growth curve of HCmel12 cells in wildtype (WT; n = 9) and nidogen-1 knockout (KO, n=10) mice. **(B)** Quantification of lung metastases from orthotopic model. WT; n = 7 mice and KO; n=10 mice. **(C)** Quantification of the size of lung metastases from tail vein injection model. WT; n = 8 mice and KO; n=7 mice. There were 6 sections taken from each lung. Plotted here is the average size of lung metastases per section. **(D)** Quantification of lung metastases from tail vein injection model. WT; n = 8 mice and KO; n=7 mice. *p < 0.05 by two-tailed Student’s *t*-test. **(E)** H&E staining of HCmel12 lung metastatic tumor from tail vein injection model. Arrows highlight examples of metastatic regions.

It has been reported that nidogen-1 knockout mice do not have increased lung metastasis compared to nidogen-1 wildtype or heterozygous mice in a B16 melanoma tail vein injection model (19). To investigate this further, we obtained nidogen-1 knockout mice and switched to a melanoma model given this previous report and the strain of the mice (not syngeneic with the breast cancer models). We investigated the role of stromal-derived nidogen-1 in melanoma lung metastasis using wildtype and nodogen-1 knockout mice. Given that B16 cells do not form sufficient spontaneous metastases from orthotopic tumors, we used HCmel12 cells that naturally lack nidogen-1 expression, in orthotopic and tail vein metastasis models in both wildtype and nidogen-1 knockout syngeneic mice (**Figure 3**).

In the orthotopic model, we did not observe any difference in primary tumor growth rate between wildtype and nidogen-1 knockout mice (**Figure 3A**). However, when we analyzed spontaneous lung metastasis in these mice, 7 out of 11 (64%) wildtype mice and 10 out of 12 (83%) of nidogen-1 knockout mice developed detectable lung metastases. Although not statistically significant, there was a trend that nidogen-1 knockout mice display more lung metastases (p = 0.28, chi-square test). Mice having lung metastases did not show any difference in the numbers of metastatic foci when comparing wildtype and nidogen-1 knockout mice (**Figure 3B**).

We then performed tail vein injection of HCmel12 into both wildtype and nidogen-1 knockout mice. 8 out of 10 wildtype mice (80%) and 7 out of 9 nidogen-1 knockout mice (78%) developed detectable lung metastases. When quantifying the number of lung metastatic lesions in mice developing lung metastasis, we found that although there was no statistically significant difference in the size of metastatic foci (**Figure 3C**), there was a significant increase in the number of metastatic foci in the nidogen-1 knockout mice compared with wildtype mice (**Figure 3D**). These findings suggest a role for stromal-derived nidogen-1 in supporting metastatic colonization.

### 3.4 Nidogen-1 knockout mice have defects in the lung alveoli

The BM is known to act as a critical barrier against cancer progression. We therefore hypothesized that loss of nidogen-1 may cause BM defects in the lungs, which compromise BM barrier function, thereby increasing the ability of cancer cells to extravasate and colonize the lungs. In order to test this hypothesis, we performed electron microscopy (EM) to check the lung alveoli structures in both wildtype and nidogen-1 knockout mice.

Previous studies showed that loss of nidogen-1 causes thinning and discontinuity of the BM in brain capillaries (20), however loss of nidogen-1 does not cause BM defects in the kidneys and muscles (10).

**Figure 4:**
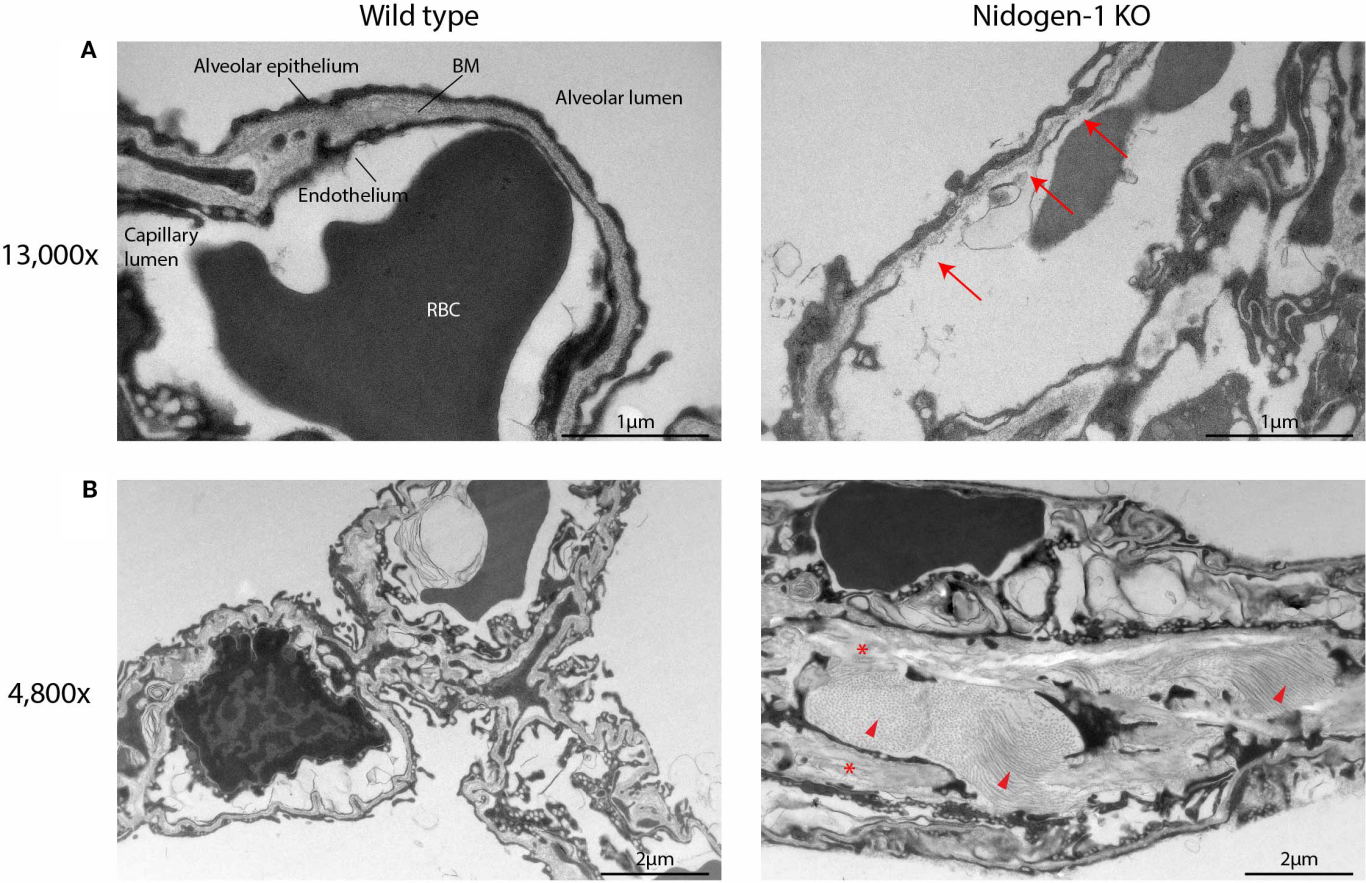
Nidogen-1 knockout mice have defects in the lung alveoli. **(A)** Representative electron microscopy (EM) images at 13,000x. BM, basement membrane. RBC, red blood cell. Arrows: fragmented endothelium with gaps, and poorly defined BM. **(B)** Representative EM images at 4,800x. Arrow heads and stars: increased amounts of fibrillar collagen and elastin, respectively. For each genotype (WT, HET, HOM) three mice were used. So, n=3 for each group, in total n=9.

Analysis of the EM data revealed defects in the lung alveoli of nidogen-1 knockout mice (**Figure 4**). First, we observed fragmented endothelium in nidogen-1 knockout mice. While lung alveoli in wildtype mice had continuous and smooth endothelium, nidogen-1 knockout mice exhibited fragmented endothelium with obvious gaps. In addition, the BM underlying the endothelium was fragmented and poorly defined in some capillaries (**Figure 4A**, arrows highlight fragmented endothelium with gaps, and poorly defined BM).

In addition, we also observed structural differences in alveolar interstitium between wildtype and nidogen-1 knockout mice. In the wildtype mouse lung, alveolar interstitium had well-defined BMs, extracellular matrix and fibroblasts, and exhibited lower amounts of fibrillar collagen and elastin. In comparison, the lungs of nidogen-1 knockout mice had enlarged interstitium with increased amounts of fibrillar collagen and elastin (**Figure 4B**, arrow heads and stars highlight increased amounts of fibrillar collagen and elastin). Moreover, we observed that in some cases red blood cells were found in the alveolar lumen instead of in capillaries in the nidogen-1 knockout mice. This suggests that the lung vessels of the nidogen-1 knockout mice are leakier.

## 4 Discussion

In this study, we first examined the expression pattern of nidogen-1. We found that one cell line of epithelial origin (the breast cancer cell line 4T1) and two cell lines of neural crest origin (the melanoma cell lines HCmel12 and B16F10) do not express nidogen-1. Moreover, we found that CAFs express high levels of nidogen-1, in agreement with previous published results (13). Similarly, it was shown that tumors formed by the human breast cancer MDA-MB-231 cell line, and by its counterpart MDA-MB-231-LM2 cell line which prefers to colonize lungs, the nidogen-1 expression is restricted to the stromal cells and not the cancer cells (21).

Using orthotopic and transgenic mouse models of breast cancer, we demonstrated that nidogen-1 expression is reduced in mammary tumors and in distant lung metastases, and that the development of CAFs may be accountable for these changes. We confirmed reduced nidogen-1 expression in healthy tissue compared with breast cancer tumors in human patient datasets, demonstrating the clinical relevance of our findings. These findings suggest that tumor cells may “educate” the surrounding stroma to reduce nidogen-1 expression, potentially in connection with fibroblast activation, at both the primary and metastatic sites.

Using HCmel12 melanoma experimental metastasis model, we found that the nidogen-1 knockout mouse has increased lung metastasis, suggesting that nidogen-1 expression at the metastatic niche may be important to prevent cancer cell colonization of the lungs. Interestingly, Mokkapati et al. showed that using B16 melanoma model, loss of stromal nidogen-2, but not nidogen-1, caused increased lung metastasis (19). This surprising difference may be attributed to the divergent interactions of these two melanoma cell lines with their respective microenvironments, or the difference in genetic backgrounds of the nidogen-1 knockout mice used in these two studies.

We analyzed the lung BM structures of nidogen-1 knockout mice using electron microscopy and observed fragmented endothelium and poorly defined BM. These newly described defects indicate compromised BM barrier function, which can explain the increased metastatic capacity of cancer cells in colonizing the lungs of nidogen-1 knockout mice.

Interestingly, previous EM studies reported an intact BM in the kidney and muscles of nidogen-1 knockout mice (10). We were able to reproduce these results (**Supplementary Figure 1**). Thus, the requirement for nidogen-1 in maintaining intact BM structures may be organ-specific, for example through dependency on other BM or ECM proteins.

Given that reduced nidogen-1 affects the integrity of vessels, one could image a scenario whereby cancer cells present at the primary or metastatic site, secret factors to reduce nidogen-1 levels in the surrounding stroma, enhancing the ability of cancer cells to cross vessels and establish metastases. It is known that solid tumors can prepare specific distant organs for metastatic colonization (23). It would be interesting to test if reduced nidogen-1 expression occurs in premetastatic niches and could therefore prepare ease-of-entry for later incoming cancer cells and investigate if these effects only occur in some organs (e.g. lungs) and not others (e.g. kidney).

Another aspect worth discussing is the role of nidogen-2 in cancer metastasis. Loss of nidogen-2 has been associated with increased lung metastasis of B16 melanoma model when injected intravenously (19). The authors demonstrated that absence of nidogen-2 resulted in compromised integrity of the BM. This may be the cause of increased lung metastasis observed in nidogen-2 knockout mice. In this study, we have shown similar BM disruption in nidogen-1 knockout mice and its association with increased lung metastasis.

In this study, we used alpha-smooth muscle actin (αSMA) as a marker for CAFs. We are aware that there are limitations due to the heterogeneity of fibroblast populations within the tumor microenvironments. Some researchers show that αSMA is not exclusively expressed by CAFs. It can also be found in normal fibroblasts. It is also a main marker of smooth muscle cells covering and supporting mature vasculature. These findings were complemented by using PDGFRα as an additional CAF marker, yielding similar results. However, ideally additional CAFs markers could be used to further support the findings in this study.

Interestingly, we noticed a downregulation of Col1α in metastatic lung fibroblasts, as occurred with nidogen-1 (**Figure 2B**). Further investigation is required, for example additional verification that tumor cells were not sorted out together with the fibroblasts.

Taken together, our findings provide novel insight into cancer-stromal interplay and uncover new roles of nidogen-1 in the regulation of cancer metastasis. We have shown that many cancer cells, both epithelial originated and neural crest originated, do not express nidogen-1. Furthermore, we have shown that loss of nidogen-1 in lungs causes BM defects that lead to leakier vessels and increased metastasis.

## Data Availability

The original contributions presented in the study are included in the article/**Supplementary Material**. Further inquiries can be directed to the corresponding author.
