# Supplementary material for: Loss of nidogen-1 causes lung basement membrane defects and increased metastasis
Source: Front Immunol. 2025 Oct 16;16:1598547. doi: 10.3389/fimmu.2025.1598547 (PMC12571852; doi:10.3389/fimmu.2025.1598547)
Supplement: Supplementary file 1 [file Image1.pdf]

Supplementary Material

1 Supplementary Figures

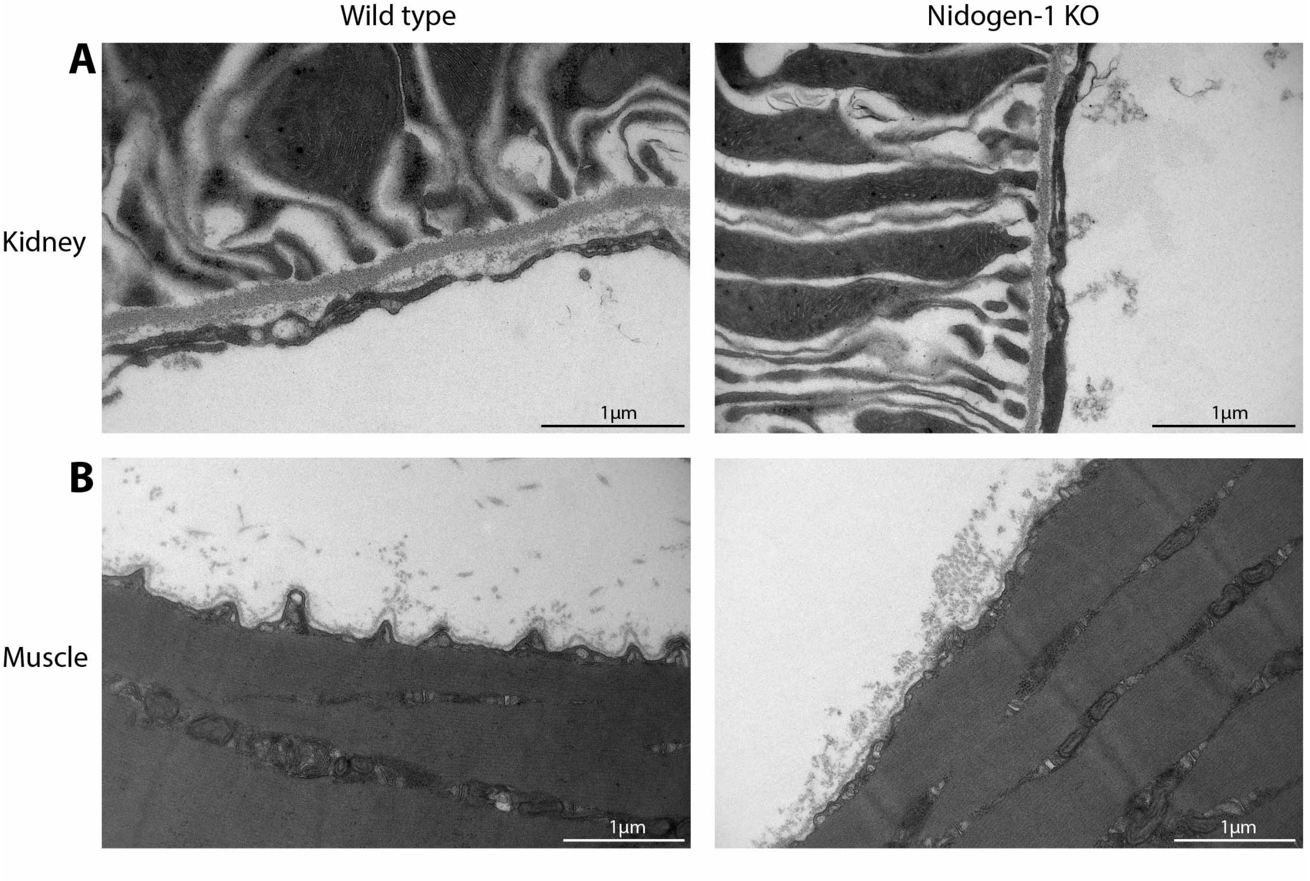

Supplementary Figure 1. Nidogen-1 knockout mice have normal BM in the kidney and muscle. (A) Representative EM images at 13,000x of kidney. (B) Representative EM images at 11,000x of muscle.

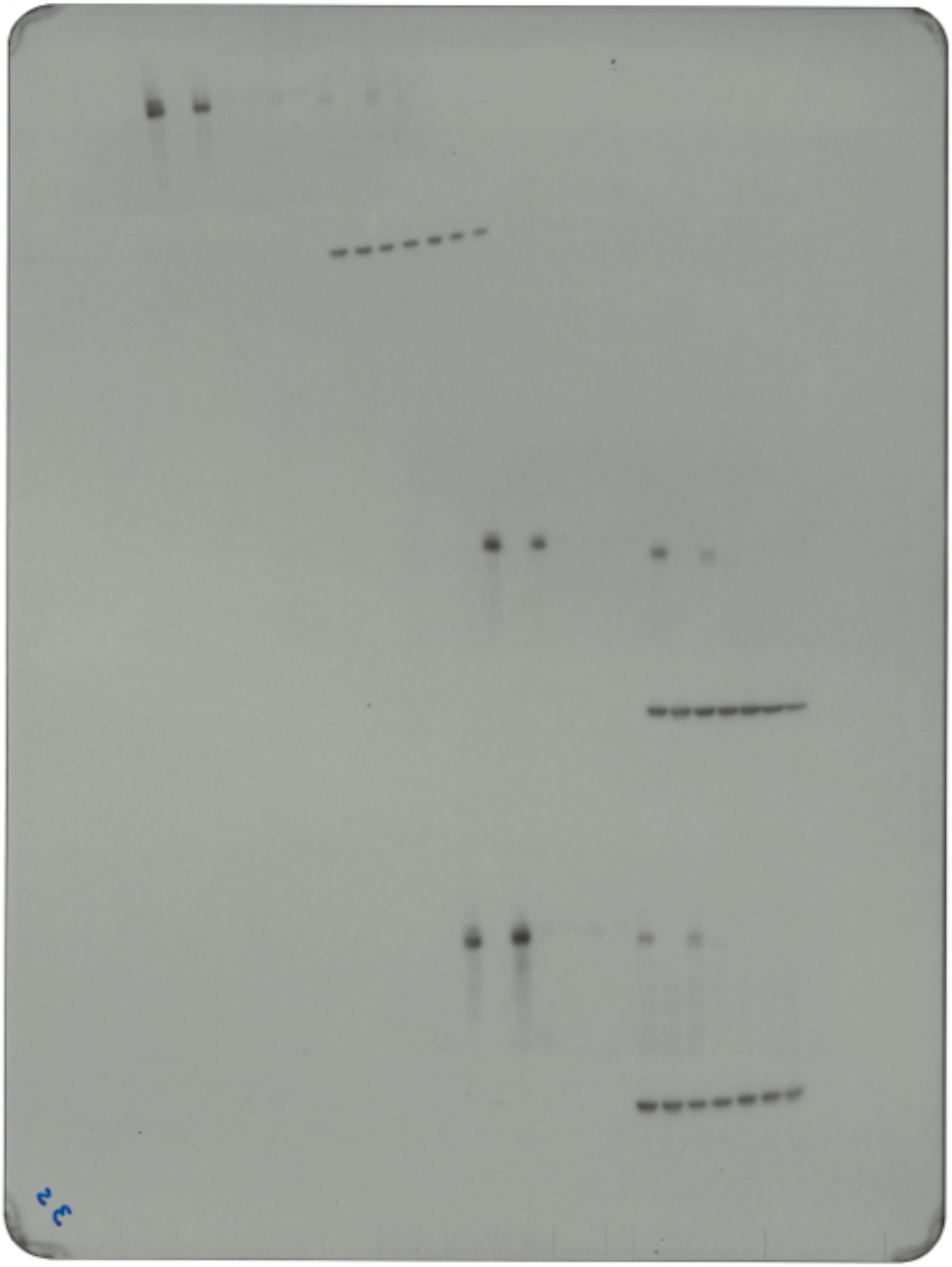

Supplementary Figure 2a. Full film of western blot in Figure 1E. Exposure time 2 seconds. Three independent biological repeats are exposed in the same film (middle one was used in Figure 1E). Lane labels are shown in Figure 1E.

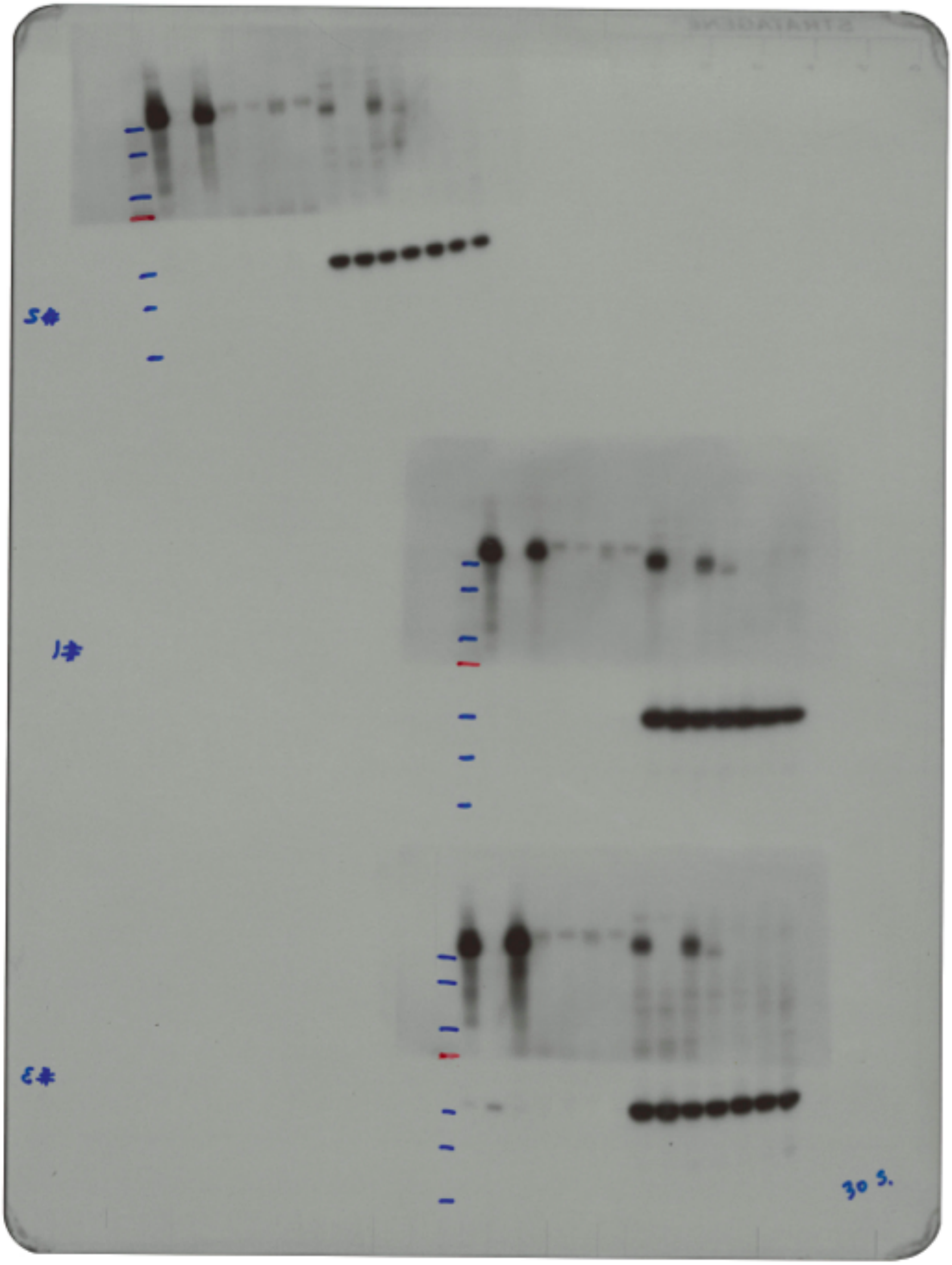

Supplementary Figure 2b. Overexposure version of Supplementary Figure 2a. Exposure time is 30 seconds. Lane labels are shown in Figure 1E. Protein size ladder from top to bottom are 170kDa, 130kDa, 100kDa, 70kDa (red), 55kDa, 40kDa, 35kDa.

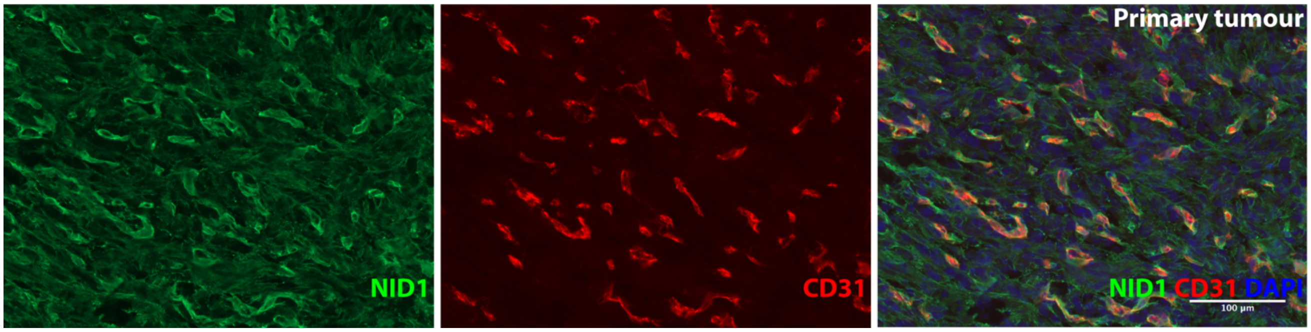

Supplementary Figure 3. CD31 (PECAM-1) and Nidogen-1 co-localisation immunofluorescent images from 4T1 primary tumour sample.

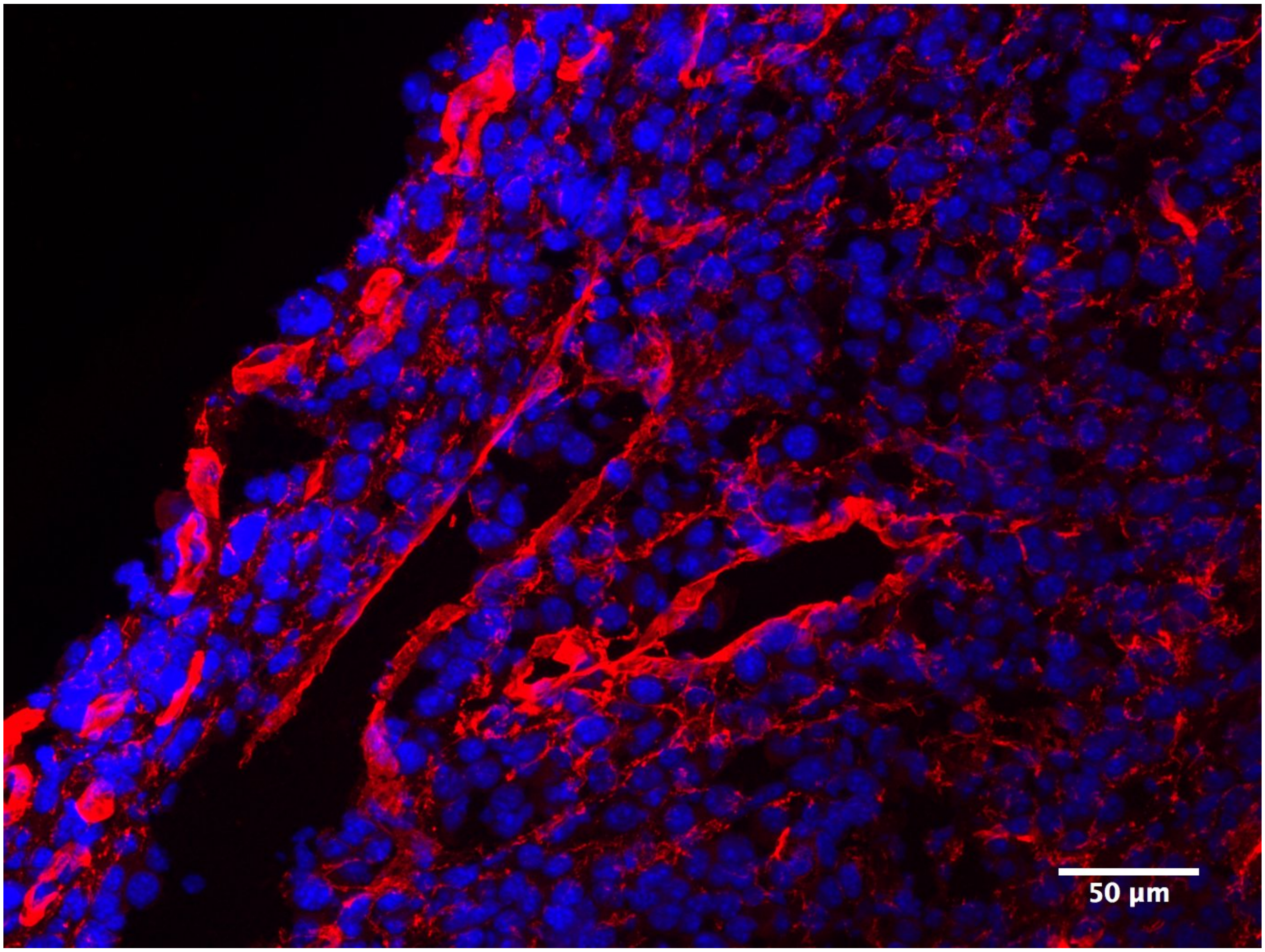

Supplementary Figure 4. Zoom in view of nidogen-1 (red) immunofluorescent staining in primary 4T1 tumour in fat pad (blue shows DAPI).

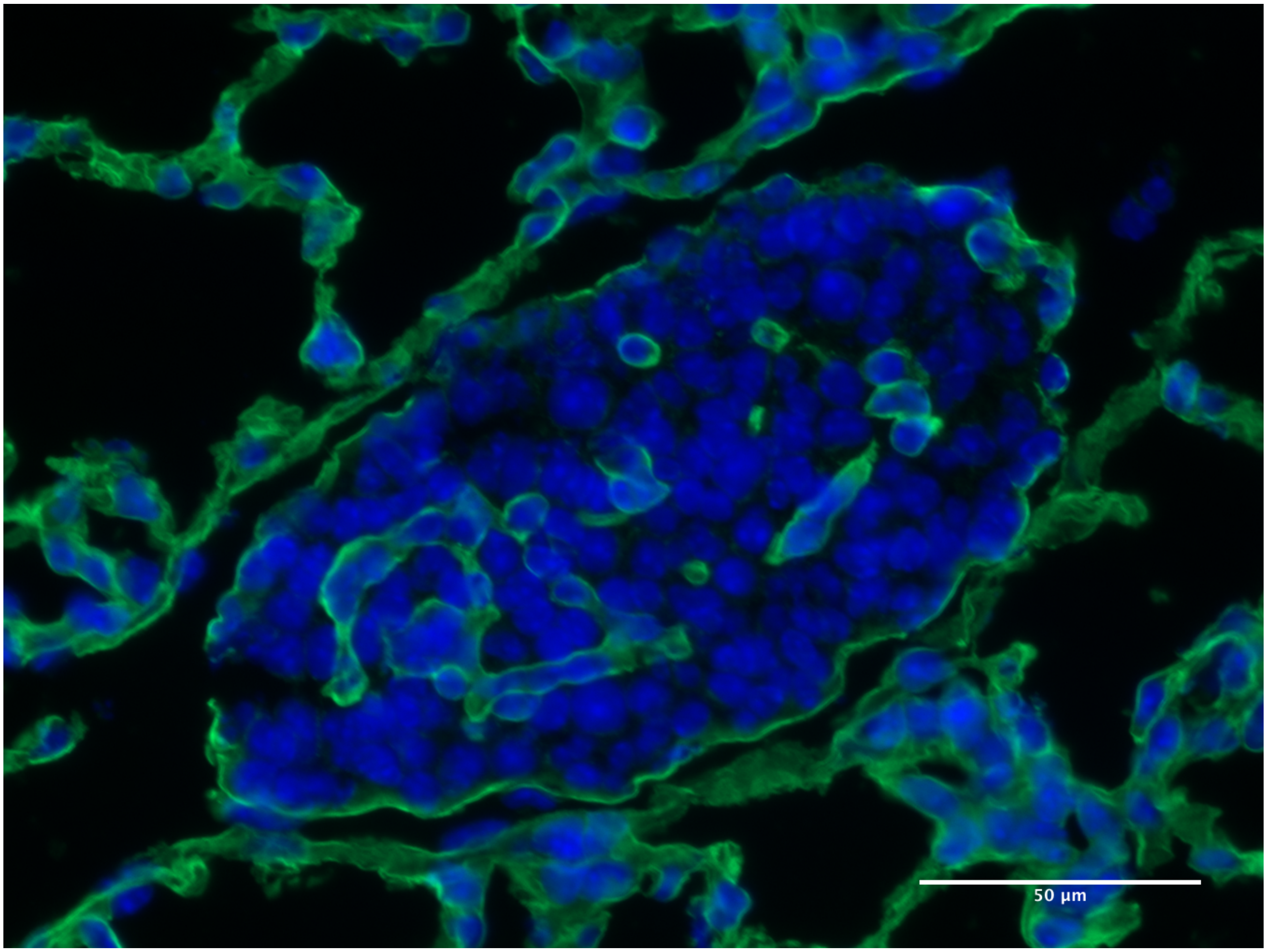

Supplementary Figure 5. Zoom in view of nidogen-1 (green) immunofluorescent staining in lung metastasis of 4T1 tumour (blue shows DAPI).

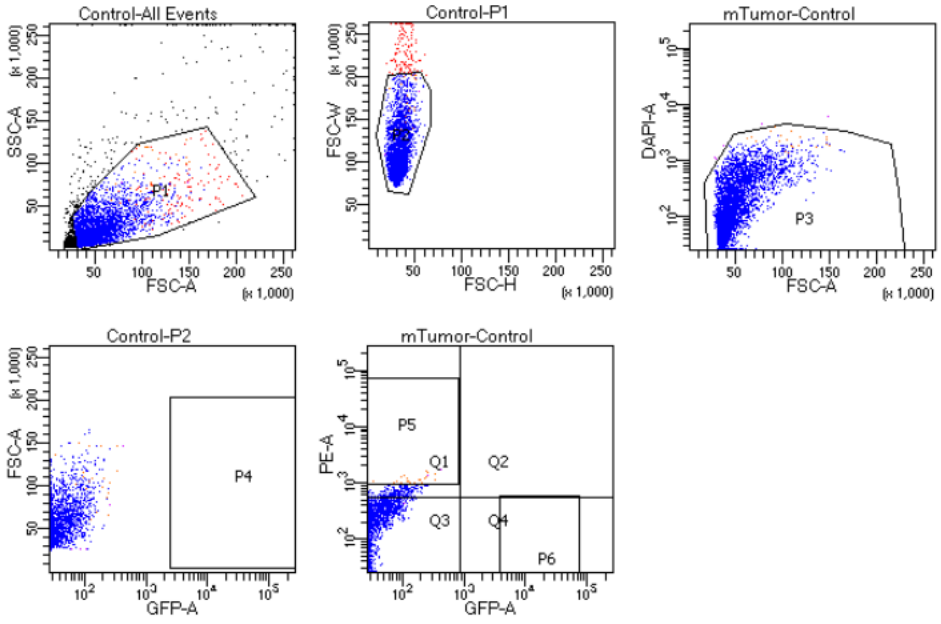

Tube: Control

Population	#Events	%Parent	%Total
All Events	10,000	####	100.0
P1	6,215	62.2	62.2
P2	6,073	97.7	60.7
P3	6,067	99.9	60.7
P4	0	0.0	0.0
Q1	232	3.8	2.3
Q2	0	0.0	0.0
Q3	5,835	96.2	58.4
Q4	0	0.0	0.0
P5	20	0.3	0.2
P6	0	0.0	0.0

Supplementary Figure 6. Tumour cell control for FACS experiment in Figure 1C. Control without colours. This is to control cells vs. debris from the sample.

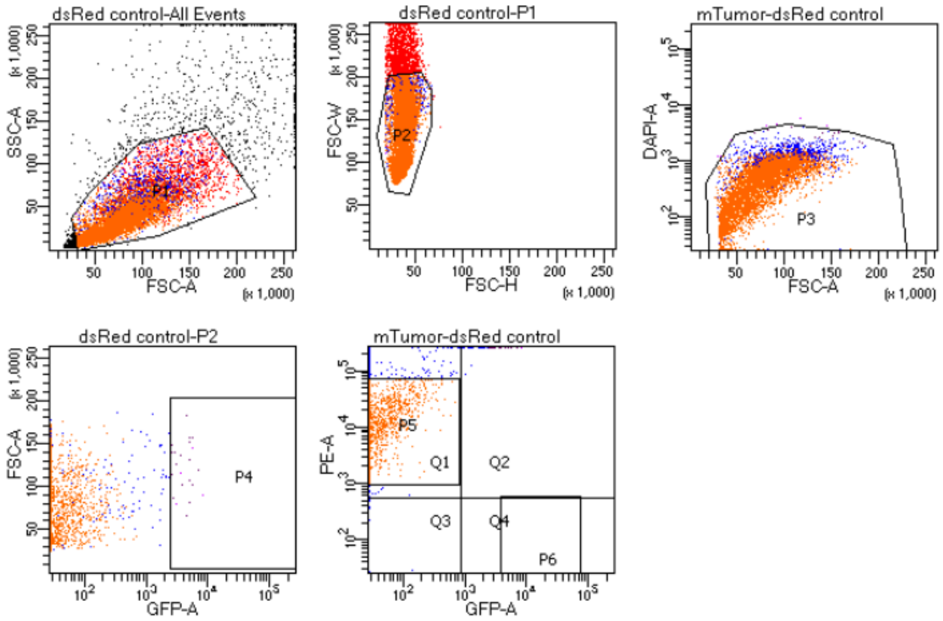

Tube: dsRed control

Population	#Events	%Parent	%Total
All Events	10,000	####	100.0
P1	8,493	84.9	84.9
P2	6,428	75.7	64.3
P3	6,421	99.9	64.2
P4	21	0.3	0.2
Q1	6,367	99.2	63.7
Q2	46	0.7	0.5
Q3	8	0.1	0.1
Q4	0	0.0	0.0
P5	5,066	78.9	50.7
P6	0	0.0	0.0

Supplementary Figure 7. dsRed channel control for FACS experiment in Figure 1C. This is a single colour control for dsRed to adjust the voltage.

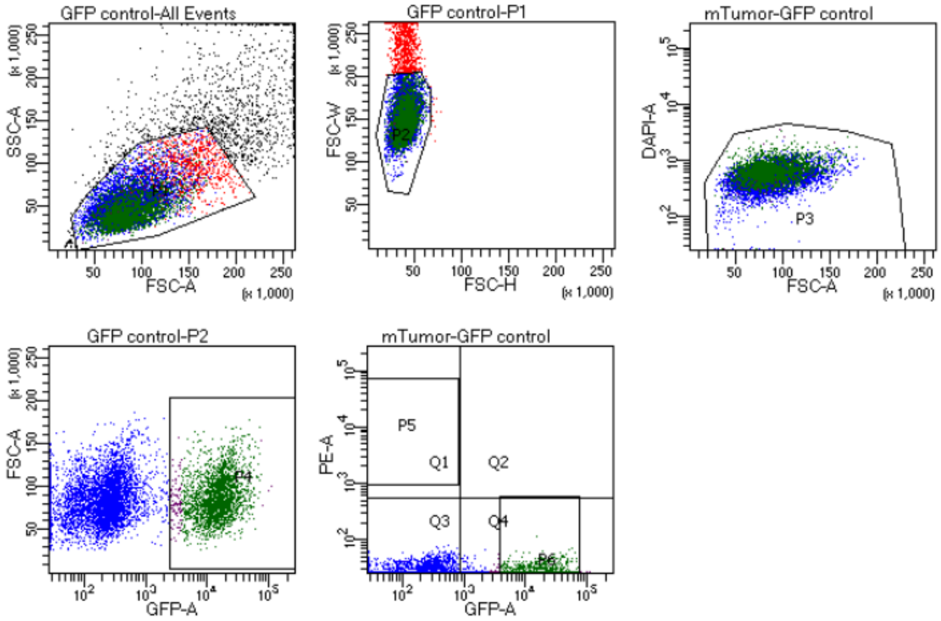

Tube: GFP control

Population	#Events	%Parent	%Total
All Events	10,000	####	100.0
P1	8,623	86.2	86.2
P2	7,482	86.8	74.8
P3	7,482	100.0	74.8
P4	2,501	33.4	25.0
Q1	0	0.0	0.0
Q2	0	0.0	0.0
Q3	4,828	64.5	48.3
Q4	2,654	35.5	26.5
P5	0	0.0	0.0
P6	2,408	32.2	24.1

Supplementary Figure 8. GFP channel control for FACS experiment in Figure 1C. This is a single colour control for GFP to adjust the voltage.

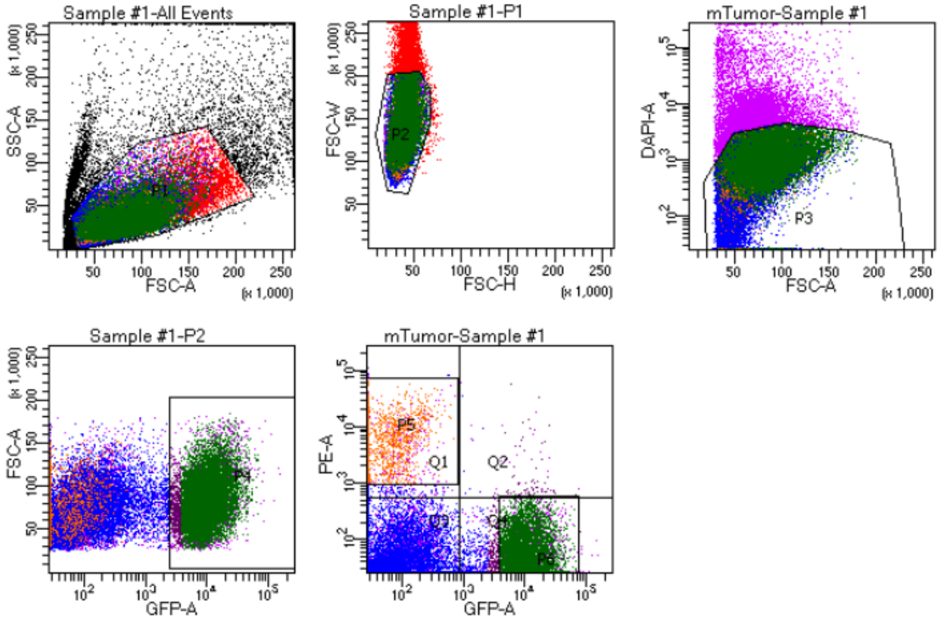

Tube: Sample #1

Population	#Events	%Parent	%Total
All Events	110,000	####	100.0
P1	75,202	68.4	68.4
P2	71,268	94.8	64.8
P3	62,267	87.4	56.6
P4	15,649	25.1	14.2
Q1	1,417	2.3	1.3
Q2	87	0.1	0.1
Q3	44,536	71.5	40.5
Q4	16,227	26.1	14.8
P5	1,291	2.1	1.2
P6	14,957	24.0	13.6

Supplementary Figure 9. Detailed view of the FACS experiment in Figure 1C. This is the experimental set up to sort out dsRed+ and GFP+ cells.

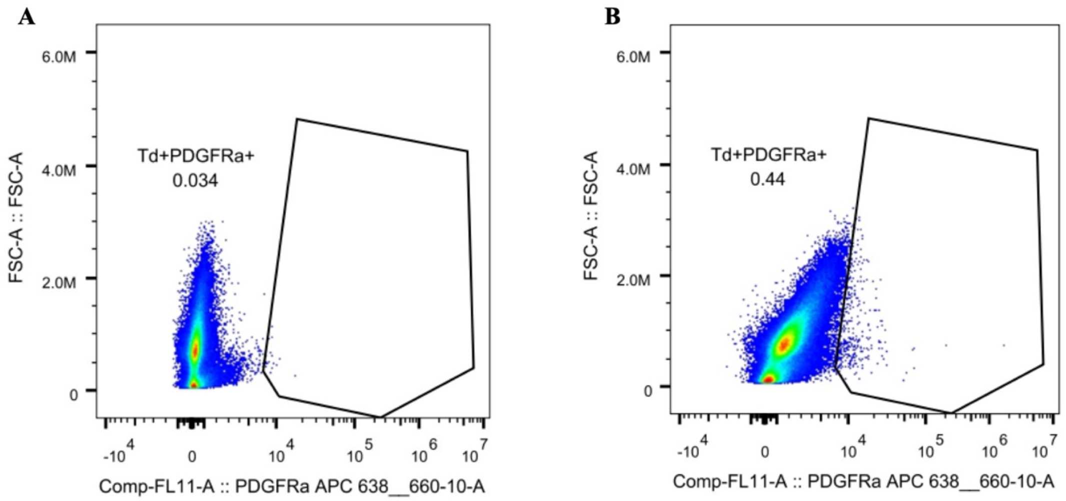

To determine whether 4T1-Td cancer cell line express PDGFRα, we performed flow cytometry using an APC-conjugated anti-PDGFRα antibody. As shown in panel **A**, the unstained control sample have minimal background signal (0.034%). Similarly, the stained sample (panel **B**) shows only a slight increase (0.44%), without a clearly distinct PDGFRα-positive population. These result indicates that 4T1-Td cells do not express PDGFRα.

*The same results now with their gating strategy

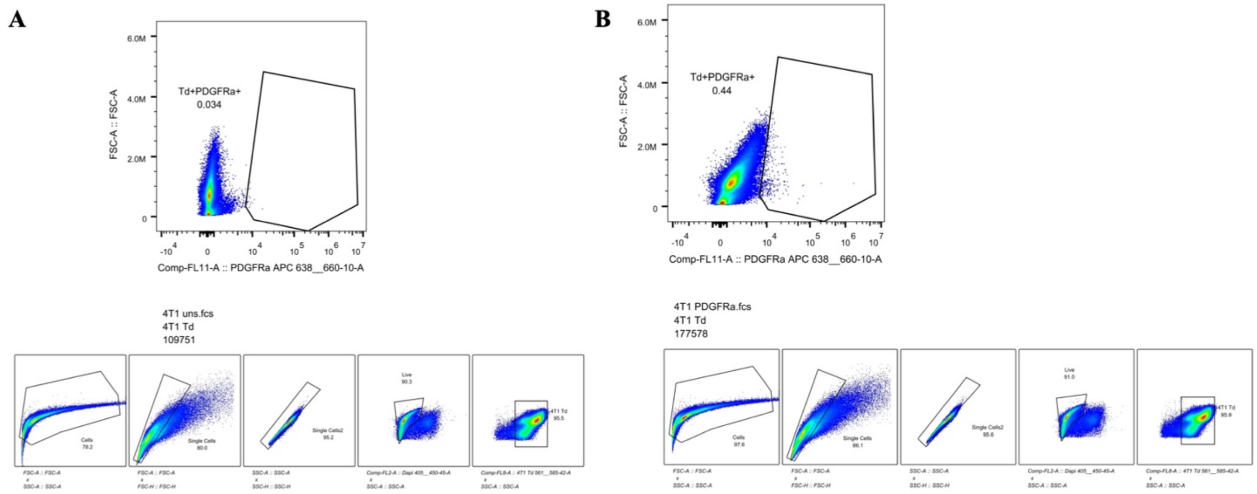

Supplementary Figure 10. 4T1 cells do not express PDGFRα
